# Effects of *Clostridium butyricum* on Growth Performance, Intestinal Health, and Disease Resistance of Hybrid Grouper (*Epinephelus fuscoguttatus♀ × E. lanceolatus♂*) Fed with Cottonseed Protein Concentrate (CPC) Replacement of Fishmeal

**DOI:** 10.1155/2023/1184252

**Published:** 2023-06-01

**Authors:** Ling Pan, Weikang Li, Ruitao Xie, Hongyu Liu, Beiping Tan, Xiaohui Dong, Qihui Yang, Shuyan Chi, Shuang Zhang

**Affiliations:** ^1^Laboratory of Aquatic Animal Nutrition and Feed, Fisheries College, Guangdong Ocean University, Zhanjiang 524088, China; ^2^Key Laboratory of Aquatic, Livestock and Poultry Feed Science and Technology in South China, Ministry of Agriculture and Rural Affairs, Zhanjiang 524088, China; ^3^Aquatic Animals Precision Nutrition and High Efficiency Feed Engineering Research Center of Guangdong Province, Zhanjiang 524088, China; ^4^Guangdong Evergreen Feed Industry Co. Ltd., Zhanjiang 524088, China

## Abstract

An 8-week feeding trial was conducted to investigate the effects of *C. butyricum* on the growth performance, microbiota, immunity response, and disease resistance in hybrid grouper fed with cottonseed protein concentrate (CPC) replacement of fishmeal. Six groups of isonitrogenous and isolipid diets were formulated including a positive control group (50% fishmeal, PC), a negative control group (CPC replaced 50% of fishmeal protein, NC), and *Clostridium butyricum* supplemented with 0.05% (C1, 5 × 10^8^ CFU/kg), 0.2% (C2, 2 × 10^9^ CFU/kg), 0.8% (C3, 8 × 10^9^ CFU/kg), and 3.2% (C4, 3.2 × 10^10^ CFU/kg), respectively, to the NC group. The results showed that weight gain rate and specific growth rate were significantly higher in the C4 group than that in the NC group (*P* < 0.05). After supplementation with *C. butyricum*, the amylase, lipase, and trypsin activities were significantly higher than the NC group (*P* < 0.05; except group C1), and the same results were obtained for intestinal morphometry. The intestinal proinflammatory factors were significantly downregulated, and the anti-inflammatory factors were significantly upregulated in the C3 and C4 groups compared with the NC group after supplementation with 0.8%-3.2% *C. butyricum* (*P* < 0.05). At the phylum level, the PC, NC, and C4 groups were dominated by the Firmicutes and the Proteobacteria. At the genus level, the relative abundance of *Bacillus* in the NC group was lower than that in the PC and C4 groups. After supplementation with *C. butyricum*, grouper in the C4 group showed significantly higher resistance to *V. harveyi* than the NC group (*P* < 0.05). Above all, taking into account the effects of immunity and disease resistance, it was recommended to supplement 3.2% *C. butyricum* in the diet of grouper fed the replacement of 50% fishmeal protein by CPC.

## 1. Introduction

Hybrid grouper (*Epinephelus fuscoguttatus♀ × E. lanceolatus♂*) is an import economical fish which is widely cultured in the coastal areas of Southeast Asia for its fast growth rate and high disease resistance [1–3]. Being a voracious carnivorous fish, hybrid grouper has a high demand for high-quality protein [4]. For this reason, fishmeal supplementation levels in diets are usually very high (50~60%) in order to ensure the growth of grouper during farming culture [5, 6]. In recent years, fishmeal, as the main raw material for aquaculture diets, has continued to rise in price with the increase in demand for aquaculture products and the further expansion of aquaculture scale [7]. Therefore, it is crucial to discover new sources of affordable and broadly accessible protein in order to lower diet prices and aquaculture costs. Our annual production of cottonseed is tremendous, and the cottonseed protein concentrate (CPC) produced from it is cheaper than fishmeal, making it a sustainable raw material for aquaculture [8]. In recent years, there has been a lot of attention on cottonseed protein concentrate (CPC) as a nonfood plant protein source [6, 9]. CPC is a high protein refined produced from cottonseed that has been stripped of its lint, shelled, steeped in oil at low temperatures in one step, drained, and then stripped of the toxic substance gossypol [10]. The protein content of CPC with very little residues of gossypol is 60%-70%, which is close to the protein content of fishmeal [6, 8]. The replacement of 45% of fishmeal by CPC did not adversely affect the growth performance of largemouth bass (*Micropterus salmoides*) [11]. Chen et al. found that the replacement of 24% of fishmeal by CPC would improve the growth performance of grouper [12]. Black tiger shrimp (*Penaeus monodon*) showed the best growth performance when CPC replaced 33.33% of fishmeal [13]. However, the growth performance of golden pomfret (*Trachinotus ovatus*) and *Litopenaeus vannamei* decreased significantly with a sharp increase in the proportion of fishmeal substituted with CPC [9, 10]. The above studies indicate that CPC can be used as a replacement for fishmeal with appropriate proportion but should not be supplemented in excessive amounts in the aquaculture diets. This is a common problem for all plant proteins. Excessive fishmeal substitution for plant protein could cause a decline in growth performance, decreased immunity, and disease resistance and produce intestinal damage in aquatic animals [12, 14–16]. Some environmentally friendly additives, like herbal extracts, exogenous enzymes, and prebiotics, have been extensively applied in aquaculture with the purpose of improving the proportion of plant protein supplementation as well as to safeguard the health of aquatic animals [17–19].

Probiotics are bioactive substances that are beneficial to the host, which regulate the intestinal microecological balance and promote intestinal digestion and absorption [20]. *Bacillus* spp. [21–23], *Lactobacillus* spp. [24, 25], *Aeromonas* spp. [26], and other single or compound probiotics are the main probiotics currently available for aquaculture. *Clostridium butyricum*, a rare probiotic in the genus Clostridium, is a Gram-positive anaerobic bacterium capable of releasing bioactive substances such as B vitamins, secreting short-chain fatty acids such as butyric acid [27], and promoting digestive enzymes such as trypsin, amylase, and lipase activities [28]. Moreover, it has the benefits of being resistant to heat, bile, and acid compared to other probiotics [29]. The ability of feed supplementation with *C. butyricum* to improve growth performance has been reported in a variety of aquatic animals. *C. butyricum* supplementation on the diet of mandarin fish (*Siniperca chuatsi*) significantly increased its survival rate and improved its intestinal microbial composition [30]. Intestinal morphology was improved and intestinal pathogenic bacteria were reduced in large yellow croaker (*Larimichthys crocea*) fed and supplemented with *C. butyricum* [31]. It was found that *C. butyricum* significantly increased the activity of digestive enzymes and regulated the intestinal microbial composition in farmed tilapia (*Oreochromis niloticus*) [32]. The addition of 1 × 10^11^ and 1 × 10^12^ CFU/kg of *C. butyricum* live cells to the feed of *Litopenaeus vannamei* significantly increased the weight gain rate and specific growth rate and significantly reduced the feed conversion ratio [33]. Feeding tilapia with *C. butyricum* (1.5 × 10^8^ CFU/g) for 8 weeks (at 0, 0.5, 1, 2, 4, and 8 g/kg) increased the weight gain rate, protein, and lipid deposition [34]. The above studies show that *C. butyricum* can improve the growth performance, protect intestinal health, and strengthen the immunity and resistance to disease of aquatic animals. However, it is unknown whether *C. butyricum* can solve the problems of reduced growth performance, low immunity, weakened disease resistance, and intestinal damage in aquatic animals caused by plant protein substitution.

The hybrid grouper is a new species of fish created by breeding female tiger groupers with male gigantic groupers. Because of its fast growth rate and commercial significance, it has been widely cultured in South China. Therefore, the objective of the present experiment was to evaluate the influence of *C. butyricum* on the growth performance, intestinal digestibility, morphology, microbiota, immunity response, and disease resistance of grouper fed with CPC instead of fishmeal.

## 2. Materials and Methods

### 2.1. Experimental Diets

Six groups of isonitrogenous and isolipid diets were formulated including a positive control group (50% fishmeal, PC), a negative control group (CPC replaced 50% of fishmeal protein, NC), and *Clostridium butyricum* supplemented with 0.05% (C1, 5 × 10^8^ CFU/kg), 0.2% (C2, 2 × 10^9^ CFU/kg), 0.8% (C3, 8 × 10^9^ CFU/kg), and 3.2% (C4, 3.2 × 10^10^ CFU/kg), respectively, to the NC group. *C. butyricum* (1 × 10^9^ CFU/g) was obtained from Bio-Form Biotechnology (Guangdong) Co., Ltd. (Foshan, China), in which the value of the bacterial content was computed from the product provided by the company. The product was included in the formulation in the form of a powder. The feed ingredients were pulverized and filtered through an 80-mesh filter, then weighed precisely, and blended in accordance with the experimental design. Using a V-type mixer (South China University of Technology, Guangzhou, China), the ingredients were homogeneously mixed step by step by adding *C. butyricum*. After that, fish oil and soy lecithin were added and mixed well again and passed through the 80-mesh sieve. Then, an appropriate amount of water was injected into the mixer, and 2.5 mm pellets were extruded with a twin-screw extruder (F-75, South China University of Technology, China). Finally, all feeds were naturally air-dried at room temperature and then stored at -20°C in plastic Ziploc bags until used. The formulation and proximate composition of the experimental diets are presented in Table 1.

### 2.2. Feeding Trial and Experimental Conditions

The feeding trials were performed in 0.3 m^3^ tanks in an indoor culture system at the Marine Biology Research Base of Guangdong Ocean University (Donghai Island, Zhanjiang, China). The experimental fish were purchased from the Hongyun Fish Hatchery (Zhanjiang, China) and temporarily reared in an outdoor concrete pond for 1 week. 540 healthy, uniform groupers with an average weight of 7.83 ± 0.01 g were randomly divided into three duplicate groups with 30 fish in each tank (0.3 m^3^). When the fish were transferred to the tanks, they were starved for a day to acclimate to the environment, followed by feeding the next day. Feed the grouper at 08:00 and 17:00 every day until evident satiety, and record the amount of food given [35]. During the 8-week feeding trial, the dissolved oxygen concentration was ≥5 mg/L, the water temperature was 28 ± 2°C, the water salinity was 28-30, and the pH was 7.8-8.2.

### 2.3. Sample Collection

A 24-hour starvation treatment was applied to the groupers at the end of an 8-week feeding trial, and then, groupers in each tank were immediately weighted and counted to calculate growth performance. After anesthesia, the middle intestine of 6 groupers per tank was used to determine intestinal digestive enzymes and intestinal immune genes. The contents of the hind intestine were collected from 6 groupers per tank for intestinal microbiome analysis. The above samples were collected and snap frozen in liquid nitrogen and then transferred to a -80°C refrigerator until analysis. The middle intestine of three groupers per tank was taken and preserved in 4% formaldehyde solution for intestinal morphology observation. The procedures involving animals and the care of animals in this study were performed in accordance with the National Institutes of Health Guidelines (NIH Pub. No. 85-23, revised 1996) and were endorsed by the Guangdong Ocean University Animal Care and Use Committee.

### 2.4. Challenge Test

The challenge test was conducted at the end of the feeding trial. After sampling, 24 fish were randomly selected from each group and placed in a tank where the fish were starved throughout the challenge test. The fish were starved during the challenge test. The *Vibrio harveyi* used in the experiment was obtained from the Key Laboratory of Control for Disease of Aquatic Economic Animals of Guangdong Higher Education Institutes (Zhanjiang, China). Each fish was injected with 0.5 ml of the bacterial solution (1 × 10^8^ CFU/ml). The specific operation method is referred to Liu et al. [36]. Fish mortality was observed every 4 hours continuously for 96 hours.

### 2.5. Growth Parameters and Biochemical Analysis

Growth performance is calculated using the following formula:

(1) $$\begin{array}{c} \text{Weight gain rate }\left(\text{WGR},\%\right)=100\times \left(\frac{\text{final weight }\left(\text{g}\right)-\text{initial weight }\left(\text{g}\right)}{\text{initial weight }\left(\text{g}\right)}\right),\\ \text{Specific growth rate }\left(\text{SGR},\%/\text{day}\right)=100\times \left(\frac{\ln\text{final weight }\left(\text{g}\right)-\text{initial weight }\left(\text{g}\right)}{\text{days}}\right),\\ \text{Survival rate }\left(\text{SR},\%\right)=100\times \left(\frac{\text{final number of fish}}{\text{Initial number of fish}}\right),\\ \text{Feed conversion ratio }\left(\text{FCR},\%\right)=\frac{\text{food intake }\left(\text{g}\right)}{\text{final weight }\left(\text{g}\right)-\text{initial weight }\left(\text{g}\right)},\\ \text{Cumulative mortality rate }\left(\%\right)=100\times \left(\frac{\text{number of dead fish in challenge experiment}}{\text{initial number of fish in challenge experiment}}\right). \end{array}$$

The routine nutrient composition of diets was determined by AOAC (1995) method [37]. The moisture content was determined by the drying constant weight method at 105°C; the crude protein content was determined by the Dumas nitrogen analyzer (Skalar, Netherlands); the crude fat content was determined by the fat analyzer (Ankom, USA); and the crude ash content was determined by the Muffle furnace burning at 550°C.

### 2.6. Intestinal Digestive Enzyme Activity Assay

In brief, intestinal samples were weighted as described previously, added 9 times the volume of saline at a ratio of 1 : 9 by weight and volume, mechanically homogenized under ice water bath conditions, and centrifuged at 2500 rpm (733 g) for 10 minutes at 4°C, and the supernatant was used to determine the activities of trypsin (TRY, ml036384), lipase (LIP, ml036371), and amylase (AMS, ml036449). The above digestive enzymes were determined using the 96T ELISA kits obtained from Meilian Biotechnology Co., Ltd. (Shanghai, China). All experiment operations were strictly carried out according to the instructions of Pan et al. [38].

### 2.7. Intestinal Morphology

The middle intestine of three groupers per tank was separated, washed in sterile saline (0.65%), and preserved in 4% formaldehyde solution for intestinal morphology fixation. Intestinal AB-PAS (Alcian blue-periodic acid Schiff) sectioning slices were prepared by Seville Biological Technology Co., Ltd. (Guangzhou, China). And afterwards, the folds and muscle layers were observed using a light microscope (Leica, DM6000), measured, and photographed using cell Sens Standard 1.8 and LAS 3.8 software, respectively. Ten fields of view of each section were randomly selected for measurement. In previous research [39], villus height, villus width, and muscle thickness have been measured as intestinal morphological metrics.

### 2.8. Real-Time Quantitative PCR Analysis

The total RNA was extracted from grouper midgut using TransZol Up (Beijing TransGen Biotech Co., Ltd., Beijing, China), and the quantity and quality of RNA samples were determined on a spectrophotometer and a 1% agarose gel [40]. According to the instructions, the extracted RNA was used for cDNA synthesis with Accurate Biology Evo M-MLV kit (Hunan, China), and the instructions were followed strictly [41]. Specific primers were designed based on published sequences of grouper (Table 2). Finally, the obtained cDNA was stored in a -20°C refrigerator for real-time quantification. All real-time PCR reaction volume of 10 *μ*l (1 *μ*l cDNA, 0.8 *μ*l primer, 3.2 *μ*l RNase Free dH_2_O, and 5 *μ*l SYBR® Green Real-Time PCR Master Mix) was conducted on a Light Cycler 480 Biosystems Real-Time PCR system (Shanghai, China). Based on the results of our preliminary experiments on the evaluation of internal control genes, *β*-actin was used as a reference gene to normalize cDNA loading. The relative gene expression results of the proinflammatory factors interleukin-4 (*il4*), interleukin-8 (*il8*), Toll-like receptor-2 (*tlr2*), and tumor necrosis factor-*α* (*tnf-α*) and the anti-inflammatory factors Toll-like receptor-1 (*tlr-1*) and tumor necrosis factor-*β* (*tnf-β*) were calculated by the 2^-*ΔΔ*CT^ method [40]. All primers were designed according to the published NCBI grouper sequences and synthesized by Sangon Biotech (Shanghai) Co., Ltd.

### 2.9. Intestinal Microbiome Analysis

The PC group, NC group, and C4 group were selected according to the experimental design and growth performance to analyze the intestinal microbial composition. The intestinal microbiota (16S V3 + V4 amplified region) of the grouper was examined using microbiome 16S rDNA sequencing with the assistance of Gene Denovo Biotechnology Ltd. (Guangzhou, China). For example, alpha diversity mainly included Chao1, observed species, Good' coverage, Shannon, Simpson, and other indicators to reflect species richness and homogeneity. According to our experimental results, we chose the unweighted_unifrac Welch's t method in PCoA. The metrics being used for analysis and the specific methods of operation are described in this document from our laboratory [42].

### 2.10. Statistical Analysis

Cumulative mortality rates were tested using the Gehan-Breslow-Wilcoxon test, with “^∗^” representing significant differences (*P* < 0.05). Other data were checked for homogeneity of variance by Levene's test and, if necessary, transformed by arcsine function. Results were subjected to one-way ANOVA using IBM SPSS Statistics software (SPSS version 26.0, Inc., Chicago, IL, USA) followed by Tukey's multiple range test to determine significant differences among treatment groups at the level of *P* < 0.05. Results were expressed as mean ± SD (standard deviation), and *n* = 3 was applied to all data except for challenge test (*n* = 3).

## 3. Result

### 3.1. Growth Performance

As shown in Table 3, a significant reduction in WGR and SGR was observed in the NC group compared with the PC group (*P* < 0.05). With the increase of dietary *C. butyricum*, the WGR and SGR tended to increase, reaching a maximum in the C4 group, which was significantly higher than that in the NC group (*P* < 0.05). There was no significant difference in SR and FCR among the groups (*P* > 0.05).

### 3.2. Intestinal Digestive Enzyme Activity

As shown in Table 4, the AMS, LIP, and TRY activities in the NC group were significantly lower than those in the PC group (*P* < 0.05). The TRY activity tended to rise with the increase of dietary *C. butyricum* and was observed to be at its maximum in the C4 group, which was significantly higher than that in the NC group (*P* < 0.05). The AMS activity went up with the increase of dietary *C. butyricum* and reached a maximum value in the C3 group, which was significantly higher than that in the NC group (*P* < 0.05). No significant difference in LIP activity was found in all groups supplemented with *C. butyricum* (*P* > 0.05) but was significantly higher than that in the PC and NC groups (*P* < 0.05).

### 3.3. Intestinal Morphology Observation

**Figure 1:**
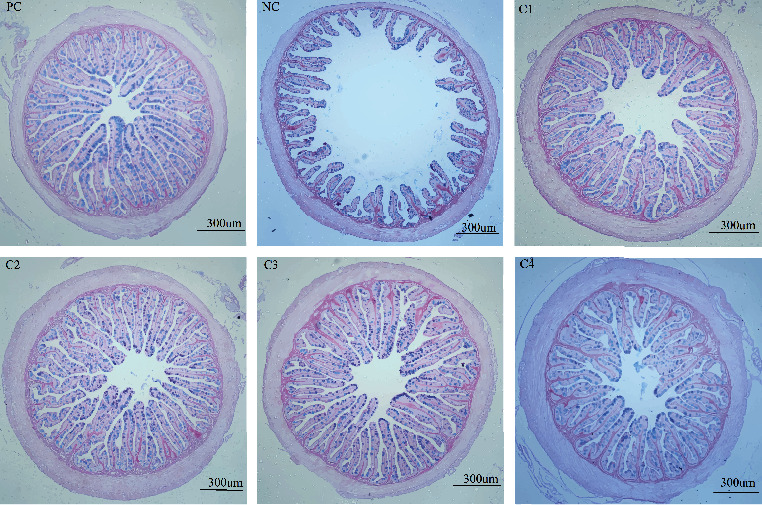
Effects of dietary supplementation with *C. butyricum* on the intestinal morphology of grouper. Values with different superscripts were significantly different (*P* < 0.05).

As shown in Table 5, the villus height, villus width, and muscle thickness were significantly lower in the NC group compared with the PC group (*P* < 0.05). After *C. butyricum* supplementation, the villus height and muscle thickness reached a maximum value in the C3 and C4 groups, respectively, which were significantly higher than those in the NC group (*P* < 0.05); and the villus width was significantly higher in all groups supplemented with *C. butyricum* than that in the NC group (*P* < 0.05).  Figure 1 visually shows the intestinal morphology of grouper in all treatment groups.

### 3.4. Relative Gene Expression Involved in Immune Response

**Figure 2:**
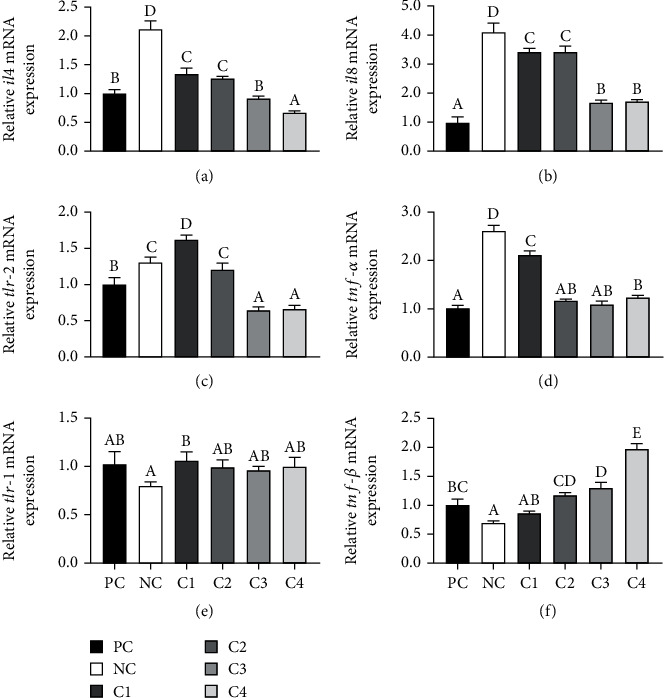
Effects of dietary supplementation with *C. butyricum* on the relative mRNA levels of intestinal immune-related genes in grouper. Values with different superscripts were significantly different (*P* < 0.05).

Compared with the PC group, the relative expression of the proinflammatory factors *il4* (Figure 2(a)), *il8* (Figure 2(b)), *tlr-2* (Figure 2(c)), and *tnf-α* (Figure 2(d)) mRNA was significantly upregulated, and the relative expression of the anti-inflammatory factor *tnf-β* (Figure 2(f)) mRNA was significantly downregulated in the NC group (*P* < 0.05). After *C. butyricum* supplementation, the relative expression of *il4*, *il8*, and *tnf-α* mRNA was significantly downregulated (*P* < 0.05), and the expression of *tnf-β* mRNA was upregulated compared with the NC group (*P* < 0.05); the relative expression of *tlr-1* (Figure 2(e)) and *tlr-2* mRNA reached a maximum in the C1 group and was significantly higher than the NC group (*P* < 0.05).

### 3.5. Microbiota Richness and Diversity Analysis

**Figure 3:**
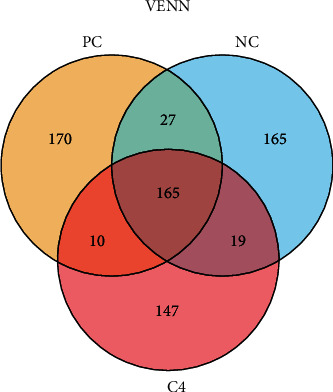
Venn diagram of OTUs in the PC, NC, and C4 groups. Venn graph was drawn by analyzing the common and unique OTUs among different groups according to the results of OTU-clustered analysis with 97% identification based on 16S rRNA sequencing data.

As shown in Figure 3, there were 165 shared OTUs in the PC, NC, and C4 groups, which accounted for 44.35%, 43.88%, and 48.39% of the PC, NC, and C4 groups, respectively. Furthermore, the microbiota coverage of all groups was close to 1, which indicates that the sequencing depth of this intestinal microbiota analysis was adequate. There were no significant differences in Chao1, ACE, Simpson, and Shannon indices among the groups (Table 6) (*P* > 0.05), indicating that there were no significant changes in grouper intestinal microbial richness and diversity in the PC, NC, and C4 groups.

### 3.6. Microbial Community Structure Analysis

**Figure 4:**
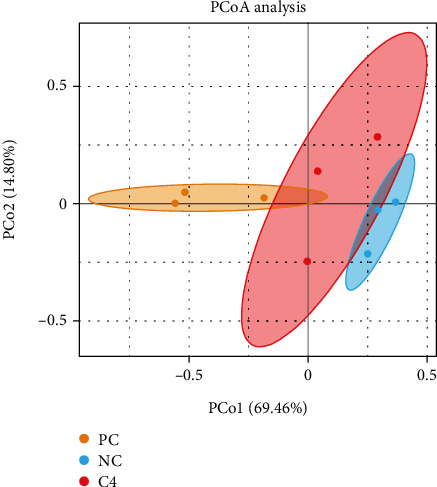
Principal coordinate analysis (PCoA) of microbial communities in grouper.

**Figure 5:**
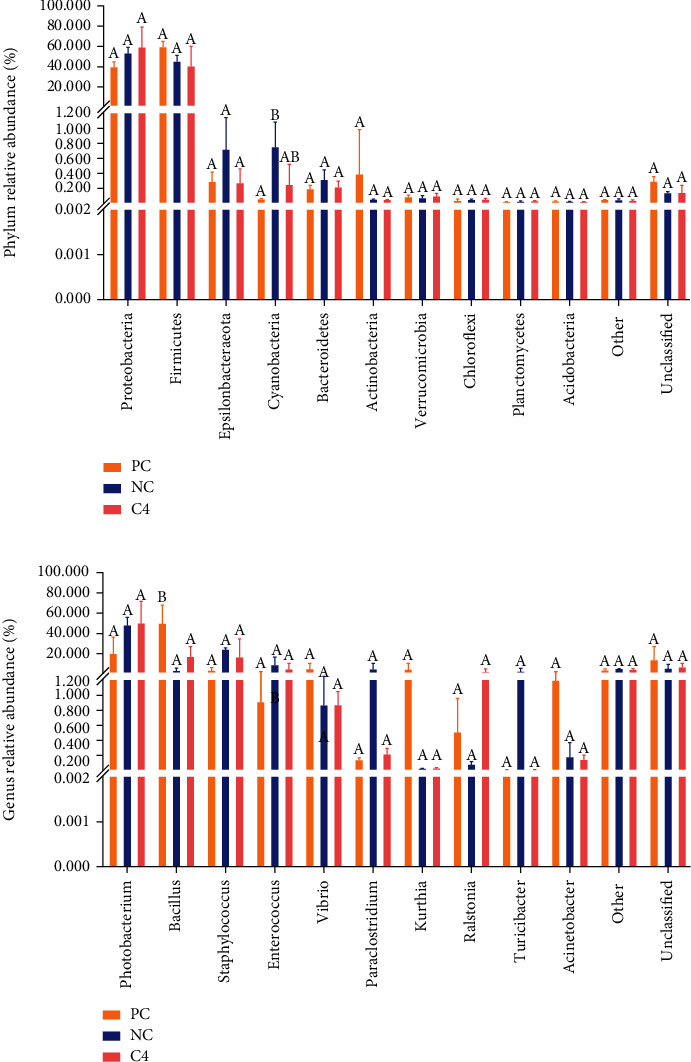
Microbial community structure of different groups. (a) Microbial community structure at the phylum level in the PC, NC, and C4 groups. (b) Microbial community structure at the genus level in the PC, NC, and C4 groups. Values with different superscripts were significantly different (*P* < 0.05).

PCo1 = 69.46%; PCo2 = 14.80%; the sum of the two was much higher than 50% (Figure 4), indicating that the analysis of differences in intestinal microbial composition is reliable. Different distance differences existed between the PC, NC, and C4 groups, indicating variability in the microbiota structure of the different groups. At the phylum level (Figure 5(a)), the PC, NC, and C4 groups were mainly dominated by the Firmicutes and the Proteobacteria. The abundance of Cyanobacteria in the PC group was significantly lower than that in the NC group (*P* < 0.05), but not significantly different from that in the C4 group (*P* > 0.05). At the genus level (Figure 5(b)), the top 10 bacteria in the three groups were *Photobacterium*, *Bacillus*, *Staphylococcus*, *Enterococcus*, *Vibrio*, *Paraclostridium*, *Kurthia*, *Ralstonia*, *Turicibacter*, and *Acinetobacter*. The abundance of *Photobacterium* in the PC group was lower than that in the NC and C4 groups (*P* > 0.05). The abundance of *Bacillus* in the NC group and C4 group was lower than that in the PC group (*P* < 0.05), while the abundance of *Staphylococcus* and *Enterococcus* was higher than that in the PC and C4 groups (*P* > 0.05).

### 3.7. Challenge Test

**Figure 6:**
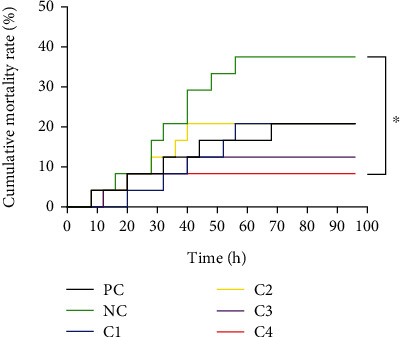
Effects of dietary supplementation with *C. butyricum* on cumulative survival of grouper. Significant differences in cumulative mortality levels between all groups were analyzed by the Gehan-Breslow-Wilcoxon test. “^∗^” indicates a significant difference (*P* < 0.05).

The death of grouper in each group by *V. harveyi* infection was concentrated before the 70th hour, and no death of grouper was found after 70 hours (Figure 6). The NC group had the highest cumulative mortality rate (37.5%) but was not significantly different from the PC group (20.83%) (*P* > 0.05). Cumulative mortality rate after *C. butyricum* supplementation reached its lowest value in the C4 group, which was significantly lower than in the NC group (*P* < 0.05).

## 4. Discussion

The growth performance of aquatic animals depends not only on whether the diet is nutritious and conducive to their digestion and absorption [43]; but the strength of the aquatic animal's own digestive and absorption capacity is also a critical factor [44]. In this experiment, the WGR and SGR of the NC group were significantly lower than those of the PC group when 50% of fishmeal protein was replaced by CPC. This may be because the application of fishmeal substitutes is often limited by the antinutritional factors, the imbalance of essential amino acids or essential fatty acids in plant proteins, and poor palatability, especially for carnivorous fish [45]; meanwhile, plant protein adversely affected its intestinal morphology, resulting in a reduced digestion and absorption of nutrients [12]. When supplemented with 3.2% *C. butyricum*, the SGR and WGR of grouper in the C4 group were significantly higher than those in the NC group, which were not significantly different from those in the PC group. This was because *C. butyricum* and its metabolites produced by various activities such as colonization, growth, and reproduction in the intestine improve the intestinal health of aquatic animals [46]. The intestinal health includes the following aspects: digestive enzymes, morphology, microbial composition, and inflammatory response of the intestine.

Various digestive enzymes in the intestines of fish facilitate the breakdown of carbohydrates, fats, and proteins from large molecules to small molecules and play an important role in the digestion and absorption of diets [47]. Digestion and nutrient absorption can be impaired by a deficiency in digestive enzymes. A diet supplemented with plant protein has been shown to reduce intestinal trypsin activity in aquatic animals [48–50]. Grouper fed soy protein concentrate instead of fishmeal showed a significant reduction in intestinal amylase activity [51]. A significant reduction in intestinal amylase and lipase activity was observed when soybean meal was substituted for fishmeal in gibel carp (*Carassius auratus*) [52]. Similar in this experiment, it was found that intestinal amylase, lipase, and trypsin activities were significantly lower in the NC group compared to the PC group. The reason for this is that plant proteins contain antinutritional factors, such as trypsin inhibitors, phytic acid, and cotton phenols, which inhibit trypsin activity in the intestine [53]. Grouper's digestive and absorption capacity was further weakened by the excessive substitution of fishmeal by CPC, which also caused severe intestinal damage and reduced amylase, trypsin, and lipase secretions. With the increase of *C. butyricum* supplementation, the intestinal digestive enzyme activities of grouper showed an increasing trend. When the supplementation amount reached 0.8%-3.2%, the intestinal amylase and trypsin activities of C3 and C4 groups were significantly higher than those in the NC group and close to those in the PC group; lipase activity was even significantly higher than that in the PC group. This is consistent with the results of feeding *L. vannamei* a diet supplemented with *C. butyricum*, which showed significantly higher digestive enzyme activity (amylase, trypsin, and lipase) [54]. This may be because *C. butyricum* can stimulate the intestine to produce more digestive enzymes [46]; furthermore, *C. butyricum* also secrete digestive enzymes such as amylase, lipase, and trypsin [55].

Aquatic animals use their intestines not only for digestion and absorption of nutrients but also for immunity [56]. Cytokines play an important role in intestinal immunity [57]. Proinflammatory and anti-inflammatory factors regulate the intestinal inflammatory response [58]. Available studies have found that grouper fed CPC instead of fishmeal triggered an intestinal inflammatory response, resulting in a significant upregulation of proinflammatory factors and a significant downregulation of anti-inflammatory factors [6, 42]. In this experiment, the relative expressions of proinflammatory factors *il4*, *il8*, *tlr-2*, and *tnf-α* mRNA were significantly upregulated, and anti-inflammatory factors *tnf-β* mRNA were significantly downregulated in the NC group compared to the PC group. This is because excessive CPC caused intestinal damage and triggered intestinal inflammatory response [42]. However, proinflammatory factors were significantly downregulated, and anti-inflammatory factors were significantly upregulated in C3 and C4 groups supplemented with 0.8% and 3.2% *C. butyricum*. This suggests that *C. butyricum* regulates the intestinal inflammatory response of grouper and improves its immunity. This is consistent with the results of a significant increase in immunity in tilapia and gibel carp fed with *C. butyricum* [59, 60]. This may be because short-chain fatty acids such as butyric acid and other fatty acids secreted by *C. butyricum* repair intestinal damage, reduce intestinal inflammation, and improve intestinal immunity [46].

A healthy and intact gut is a prerequisite for proper digestion and absorption and immunity in aquatic animals. Intestinal morphology, such as villus height, villus width, and muscle thickness, provides a visual indication of the integrity and health of the intestine [61]. The villus height and villus width determine the intestinal absorption area, and the muscle thickness determines the intestinal absorption efficiency [62]. In addition, the morphological structure of the intestinal mucosa is also associated with the function of the mucosal barrier and the inflammatory response of the intestinal mucosa [63, 64]. The NC grouper in this experiment had short intestinal villi, thinning of the muscle thickness, and severe intestinal damage. In our laboratory, excessive substitution of CPC for fishmeal caused intestinal damage in grouper in previous studies [12]. As a result of a damaged intestinal tract, its functions are impaired, resulting in reduced digestion and absorption abilities, intestinal inflammation, and a decrease in grouper growth performance and immunity. Significant improvements in villus height, villus width, and muscle thickness were observed in the grouper intestine of the experimental groups supplemented with *C. butyricum* compared to the NC group, with a significant reduction in damage. This is consistent with the results of a diet supplemented with *C. butyricum* in shrimp [33]. This is due to the ability of *C. butyricum* to produce short-chain fatty acids such as butyric acid and others, and increasing the expression of cell cycle proteins and cell proliferation nuclear antigens in epithelial cells is a way by which butyric acid promotes proliferation and differentiation, while enhancing intestinal barrier integrity by promoting the repair of the mucosal barrier and increasing the expression of mucosal barrier proteins [55].

The intestinal microbiota plays a significant role not only in promoting intestinal digestion and absorption of nutrients but also in promoting the development and regulation of the immune system [30]. Intestinal microbiota can synthesize amino acids and vitamins, participate in carbohydrate and protein metabolism, and promote the absorption of trace elements such as calcium and iron [65]. In addition, gut microbes are one of the major components of the intestinal biological barrier, and they maintain the integrity and function of the intestinal barrier by interacting with host intestinal epithelial cells, immune cells, and food residues and can influence barrier function through changes in microbial diversity [66]. On the one hand, intestinal microbiota can inhibit the growth and reproduction of pathogenic bacteria by competing for living space and nutrients [67]; on the other hand, intestinal microbiota can enhance the intestinal biological barrier function by producing metabolites such as short-chain fatty acids [46]. In the NC group, *Bacillus* was less abundant than in the PC and C4 groups based on genus analysis. This is consistent with the previous results that showed an increase in the relative abundance of *Bacillus* in the intestine of shrimp fed diets supplemented with *C. butyricum* [54]. *Bacillus* has the facility to secrete digestive enzymes and degrade macronutrients, which acts to boost intestinal digestibility in fish [68, 69]. Meanwhile, *Bacillus* can inhibit the growth and reproduction of pathogenic bacteria [70]. *Staphylococcus* and *Enterococcus* are two pathogenic bacteria that can cause an inflammatory response [71, 72]. The abundance of these two pathogenic bacteria was higher in the NC group than that in the PC and C4 groups. The above results showed that *C. butyricum* could improve the intestinal microbial composition, facilitate the abundance of beneficial bacteria, and decrease the abundance of pathogenic bacteria [59]. This is an important reason why the growth performance and immunity of grouper in the C4 group were significantly higher than those in the NC group and close to those in the PC group in this experiment.

In fish, resistance to pathogenic bacteria is closely related to their own immunity. Studies have shown that a high percentage of CPC supplementation in the diet can reduce the immunity of grouper, further leading to a reduction in disease resistance [6]. This is consistent with the results of this experiment, which is due to the fact that excessive CPC caused intestinal damage and triggered an inflammatory response in aquatic animals, reducing their resistance to exogenous pathogenic bacteria [16]. In previous studies, *C. butyricum* was found to inhibit the growth and colonization of pathogenic bacteria *Salmonella enterica* and *Vibrio parahaemolyticus* in the intestinal tract of carp [73], with high antagonistic effects against *Aeromonas hydrophila* and *Vibrio anguillarum* [67]. Shrimps fed a diet supplemented with *C. butyricum* can improve their resistance to *Vibrio parahaemolyticus* [33, 74].

A diet supplemented with *C. butyricum* significantly improves the resistance of tilapia to *Aeromonas hydrophila* [34]. In this experiment, the cumulative mortality rate of grouper injected with *Vibrio harveyi* decreased when the diet was gradually supplemented with *C. butyricum.*

Grouper mortality was significantly lower in the C4 group supplemented with 3.2% *C. butyricum* than that in the NC group. This is consistent with the results of *Litopenaeus vannamei* fed on diets supplemented with *C. butyricum*, showing a significant increase in disease resistance. In conclusion, *C. butyricum* supplementation in the diet can significantly increase aquatic animals' resistance to pathogenic bacteria. On the one hand, *C. butyricum* and its metabolites can improve the biological barrier function of aquatic animals' intestines and inhibit the colonization and growth of pathogenic bacteria [73]; on the other hand, *C. butyricum* can regulate the immune response of aquatic animals, enhance phagocytic activity, and improve immunity [75].

## 5. Conclusion

This experiment showed a reduction in growth performance, intestinal digestive enzyme activity, and immunity as well as the appearance of intestinal damage in grouper when 50% of fishmeal protein was replaced with CPC. And supplementing 3.2% (3.2 × 10^10^ CFU/kg) *C. butyricum* in diets can improve the growth performance of grouper, repair intestinal damage, increase intestinal digestive enzyme activity, enhance immunity, improve disease resistance, and improve intestinal microbial composition. Therefore, taking into account the effects of immunity and disease resistance, it was recommended to supplement 3.2% (3.2 × 10^10^ CFU/kg) *C. butyricum* in the diet of grouper fed the replacement of 50% fishmeal protein by CPC.

## Figures and Tables

**Table 1:** Composition and nutrient levels of experimental diets (dry matter, %).

Ingredient	PC	NC	C1	C2	C3	C4
Fishmeal	50.00	25.00	25.00	25.00	25.00	25.00
Concentrated cottonseed protein	0.00	26.35	26.35	26.35	26.35	26.35
Corn starch	12.00	12.00	12.00	12.00	12.00	12.00
Soybean meal	5.00	5.00	5.00	5.00	5.00	5.00
Corn gluten meal	10.00	10.00	10.00	10.00	10.00	10.00
Peanut meal	6.00	6.00	6.00	6.00	6.00	6.00
Fish oil	2.50	4.45	4.45	4.45	4.45	4.45
Soybean lecithin	2.50	2.50	2.50	2.50	2.50	2.50
Premix^1^	1.00	1.00	1.00	1.00	1.00	1.00
Choline chloride	0.50	0.50	0.50	0.50	0.50	0.50
V c	0.10	0.10	0.10	0.10	0.10	0.10
Ca(H_2_PO_4_)_2_	1.00	1.00	1.00	1.00	1.00	1.00
Antioxidant	0.05	0.05	0.05	0.05	0.05	0.05
CMC-Na	0.5	0.5	0.5	0.5	0.5	0.5
MCC	8.85	4.82	4.77	4.62	4.02	1.62
Methionine	0.00	0.23	0.23	0.23	0.23	0.23
Lysine	0.00	0.49	0.49	0.49	0.49	0.49
*Clostridium butyricum*	0.00	0.00	0.05	0.20	0.80	3.20
Total	100	100	100	100	100	100
Nutrient content^2^						
Crude protein	45.20	44.96	45.18	44.42	44.80	44.48
Crude lipid	9.13	8.76	8.66	8.96	9.10	8.82
Ash	9.00	7.30	7.30	7.50	11.90	9.40
Moisture	7.30	8.30	8.40	7.50	8.00	8.50

^1^Premix refers to a multidimensional multimineral mixture 1 : 1. (1) Vitamin premix (mg/kg diet): thiamin, 25 mg; riboflavin, 45 mg; pyridoxine-HCl, 20 mg; vitamin B12, 0.1 mg; vitamin K3, 10 mg; inositol, 800 mg; pantothenic acid, 60 mg; folic acid, 20 mg; niacin acid, 200 mg; biotin, 1.20 mg; retinol acetate, 32 mg; cholecalciferol, 5 mg; alpha-tocopherol, 120 mg; microcrystalline cellulose, 2,161.7 mg. (2) Mineral premix (mg/kg diet): Na F, 2 mg; KI, 0.8 mg; Co Cl_2_.6H_2_O (1%), 50 mg; Cu SO_4_.5 H_2_O, 10 mg; Fe SO_4_·H_2_O, 80 mg; Zn SO_4_.H_2_O, 50 mg; Mn SO_4_·H_2_O, 60 mg; Na_2_Se O_3_ (1%), 100 mg; Mg SO_4_.7H_2_O, 1,200 mg; Na Cl, 100 mg; microcrystalline cellulose, 1,347.2 mg. ^2^Values of crude protein and crude lipid contents were measured.

**Table 2:** Nucleotide sequences of the primers used to assay gene expressions by real-time PCR.

Name	Forward primer (5′-3′)	Reverse primer (3′-5′)	Product size	GenBank accession no.
*β-*Actin	CTCTGGGCAACGGAACCTCT	GTGCGTGACATCAAGGAGAAGC	146	AY 510710.2
*il4*	GAGGGCATCAGAGGAGGGAAGAG	TGGAGTCATCAGAGCAGGTGTAGG	127	XP_018557222.1
*il8*	CTCCAGACCACCACAGAGCATTG	CATCACCCACAAGAGAGCAGGATTG	96	XP_019955897.1
*tlr-2*	ACGCCATTGAGAAGAGTCACAGAAC	TAGGCTCCAACAGAATCAGCACAAC	147	AIS23534.1
*tnf-α*	CGCTGAGGAGCATCTGGTATTGTG	GCGGAGTCCTGTGGAGGGTATC	140	ADZ99105.1
*tlr-1*	CGCCAGGTCAGTATCTAGGGTAGC	GGTGATGGGAGCAGAGGAGGAG	111	AEB32452.1
*tnf-β*	CCTCTCCCATCGCTGTCCCATAG	GAGGATGCGTGTTGAGATGACTGAG	118	XM_033640148.1

**Table 3:** Effects of dietary supplementation with *C. butyricum* on the growth performance of grouper.

Items	Groups					
	PC	NC	C1	C2	C3	C4
Initial weight (g)	7.83 ± 0.00	7.83 ± 0.03	7.83 ± 0.00	7.84 ± 0.01	7.84 ± 0.01	7.83 ± 0.01
Final weight (g)	79.58 ± 1.07^d^	69.98 ± 0.62^ab^	69.70 ± 0.59^a^	68.54 ± 2.28^a^	73.40 ± 0.31^bc^	76.58 ± 1.56^cd^
WGR (%)	915.90 ± 13.71^d^	793.44 ± 6.67^ab^	792.06 ± 6.79^a^	774.39 ± 28.38^a^	836.44 ± 4.43^bc^	877.83 ± 20.57^cd^
SGR (%/d)	4.14 ± 0.02^d^	3.91 ± 0.01^ab^	3.90 ± 0.02^a^	3.87 ± 0.06^a^	3.99 ± 0.01^bc^	4.07 ± 0.04^cd^
SR (%)	100.00 ± 0.00	98.89 ± 1.92	96.67 ± 3.34	100.00 ± 0.00	96.67 ± 3.34	95.55 ± 3.85
FCR	0.82 ± 0.01	0.84 ± 0.03	0.89 ± 0.04	0.88 ± 0.07	0.87 ± 0.04	0.85 ± 0.05

Values with different superscripts in the same line are significantly different (*P* < 0.05) (*n* = 3).

**Table 4:** Effects of dietary supplementation with *C. butyricum* on intestinal digestive enzymes of grouper.

Items	Groups					
	PC	NC	C1	C2	C3	C4
AMS (mIU/mg.pro)	1216.18 ± 82.25^cd^	909.81 ± 87.72^a^	955.12 ± 23.66^ab^	1121.81 ± 57.31^bc^	1329.37 ± 53.06^d^	1307.28 ± 73.33^d^
LIP (mU/mg.pro)	1786.73 ± 107.17^b^	1516.19 ± 83.05^a^	2196.18 ± 83.39^c^	2156.88 ± 65.80^c^	2182.61 ± 88.63^c^	2216.47 ± 32.78^c^
TRY (U/mg.pro)	6211.37 ± 168.38^d^	3293.76 ± 264.02^a^	3732.41 ± 199.04^a^	4692.86 ± 254.26^b^	5514.75 ± 239.64^c^	6282.46 ± 268.04^d^

Values with different superscripts in the same line are significantly different (*P* < 0.05) (*n* = 3).

**Table 5:** Effects of dietary supplementation with *C. butyricum* on intestinal morphology of grouper.

Items	Groups					
	PC	NC	C1	C2	C3	C4
Villus height (*μ*m)	589.94 ± 16.68^c^	380.33 ± 17.09^a^	525.69 ± 25.80^b^	526.672 ± 19.35^b^	568.49 ± 18.64^bc^	529.27 ± 15.80^b^
Villus width (*μ*m)	88.85 ± 5.34^b^	66.49 ± 1.96^a^	87.36 ± 1.68^b^	88.70 ± 3.55^b^	86.89 ± 1.93^b^	87.80 ± 3.99^b^
Muscle thickness (*μ*m)	92.61 ± 2.81^b^	77.51 ± 4.13^a^	89.44 ± 2.61^ab^	109.20 ± 5.27^c^	99.04 ± 5.51^bc^	129.96 ± 8.26^d^

Values with different superscripts in the same line are significantly different (*P* < 0.05) (*n* = 3).

**Table 6:** Effects of dietary supplementation with *C. butyricum* on gut microbial alpha diversity in grouper.

Groups	Chao1	ACE	Shannon	Simpson	Coverage
PC	448.05 ± 66.88	480.80 ± 71.03	2.55 ± 0.66	0.67 ± 0.14	0.9992 ± 0.0001
NC	421.41 ± 54.91	449.99 ± 56.17	2.65 ± 0.38	0.71 ± 0.08	0.9994 ± 0.0001
C4	392.94 ± 58.24	418.73 ± 57.37	2.44 ± 0.55	0.66 ± 0.14	0.9994 ± 0.0001

Values without superscripts or with the same superscripts in the same column were not significantly different (*P* > 0.05) (*n* = 3).

## Data Availability

The data that support the findings of this study are available on request from the corresponding author. The data are not publicly available due to privacy or ethical restrictions.
